# Efficient scheme for hybrid teleportation via entangled coherent states in circuit quantum electrodynamics

**DOI:** 10.1038/srep26338

**Published:** 2016-06-01

**Authors:** Jaewoo Joo, Eran Ginossar

**Affiliations:** 1 Advanced Technology Institute and Department of Physics, University of Surrey, Guildford, GU2 7XH, United Kingdom; 2 School of Computational Sciences, Korea Institute for Advanced Study, Seoul 02455, South Korea

## Abstract

We propose a deterministic scheme for teleporting an unknown qubit state through continuous-variable entangled states in superconducting circuits. The qubit is a superconducting two-level system and the bipartite quantum channel is a microwave photonic entangled coherent state between two cavities. A Bell-type measurement performed on the hybrid state of solid and photonic states transfers a discrete-variable unknown electronic state to a continuous-variable photonic cat state in a cavity mode. In order to facilitate the implementation of such complex protocols we propose a design for reducing the self-Kerr nonlinearity in the cavity. The teleporation scheme enables quantum information processing operations with circuit-QED based on entangled coherent states. These include state verification and single-qubit operations with entangled coherent states. These are shown to be experimentally feasible with the state of the art superconducting circuits.

The scheme of quantum teleportation^1^ is of the essence for technological applications of quantum information processing such as quantum cryptography in multipartite quantum networks^2^,^3^,^4^ and for measurement based quantum computation^5^,^6^. In the original teleportation scheme, a qubit state with unknown parameters |*ψ*〉 = *a*|0〉 + *b*|1〉 with unknown *a* and *b*, transmitted from a sender (Alice) can be deterministically teleported to a receiver (Bob) by performing Bell-state measurement (BSM) through a bipartite entangled state, called a channel, in discrete variables (DVs). After the feed-forward of classical information, one can recover the original qubit state at the other location of the channel. The teleportation fidelity between the unknown state and the teleported state provides the characteristics of the channel compared with classical teleportation, which can be performed with no entangled channel (see details in the Section Method). Since the scheme of postselected DV teleportation has been firstly demonstrated in experiment of quantum optics^7^,^8^,^9^,^10^, teleportation schemes have also been demonstrated in other physical systems, particularly for deterministic methods in ion traps^11^,^12^, atomic ensembles^13^ and superconducting circuits^14^.

An alternative representation, called continuous-variable (CV) quantum teleportation^15^, has been in parallel investigated because a CV channel is indeed a natural resource for entanglement (e.g., a position-momentum entangled state in the Einstein-Podolsky-Rosen’s paper^16^). For example, the first demonstration of unconditional teleportation has been successfully performed with nonclassical CV states using two-mode squeezed states^17^,^18^,^19^,^20^. Quantum teleportation in CV states is essential to the schemes of CV quantum information processing, which have shown several advantages as compared with DV-qubit information processing^21^,^22^,^23^,^24^. These include for example time-frequency encoding^25^ and fault-tolerant CV quantum computing^26^,^27^,^28^.

One of the CV-qubit representations is based on Schrödinger cat states (SCS)^29^ given by the superposition of two phase-opposite coherent states^30^,^31^. A CV qubit (∝ *a*|*α*〉 + *b*|−*α*〉) can in principle encode information beyond DV qubits in circuit-QED^32^,^33^,^34^,^35^ because it is described in infinite dimension^17^,^18^,^19^,^36^,^37^,^38^,^39^. The SCSs have been created in various methods for instance photon adding and subtracting schemes in quantum optics^40^,^41^,^42^ and ion- or Rydberg atom-cavity systems^43^,^44^. Relatively larger SCSs have been very recently achieved in circuit-QED^45^. A generalized SCS with many different phases can be used to realise a qu*d*it which will be of use for hardware-efficient quantum memory^46^. A specific bipartite entangled CV state called an entangled coherent state (ECS), |*ψ*〉 = |*α*〉|*α*〉 + |−*α*〉|−*α*〉, naturally fits to be a channel state for CV teleportation. Generally it has been known as an excellent resource for quantum metrology and other quantum information processing^47^,^48^,^49^,^50^,^51^,^52^. Thus, DV-CV hybrid teleportation is not only an alternative for DV-qubit teleportation but also advantageous for practical quantum information processing in solid-state and cavity-QED systems^53^,^54^,^55^,^56^. For instance, these recent developments will lead innovative tools for quantum computing using hybrid single- and two-qubit gates^57^,^58^,^59^.

Here we develop a DV-CV hybrid teleportation scheme specifically designed to be implemented on a superconducting circuit. The scheme is physically hybrid in the sense that it teleports quantum information from a solid-state qubit to microwave photonic state. It is a core building block required for measurement-based quantum computation in superconducting circuits^5^,^6^,^28^,^60^. For instance a series of teleportations can mimick one- and two-qubit gates. The term ‘hybrid scheme’ discussed here has two-fold meaning: hybrid in the sense of DV and CV encoding as well as hybrid in the sense of superconducting qubit and microwave photonic state. An unknown qubit is prepared in a two-level superconducting qubit and the ECS is created in microwave photons inside two cavities with the help of an adjacent superconducting qubit^61^,^62^,^63^. In contrast to DV- and CV-only teleportations, we find that the teleportation fidelity depends not only on the amount of decoherence but also on the amplitude size of the ECS channel state. The creation of entanglement in photonic hybrid qubits has been very recently demonstrated in quantum optics^64^ and all photonic optical hybrid CV teleportation has been very recently performed^65^,^66^. However, a two-fold hybrid quantum teleportation has not been developed yet and this proposal addresses this challenge for circuit QED.

The paper is organised in the Results section as follows. We first examine a specific architecture and it consists of two high-Q cavities, three qubits (2C3Q) and two additional readout resonators at the edge, inspired by the existing experimental architecture^45^. We first explain the adverse influence of the residual self-Kerr effect in a cavity and how it can be significantly reduced. The proposed design is in line with state-of-the-art superconducting architecture as shown recently in^45^.

Next we describe the hybrid teleportation scheme. We investigate how to prepare the initial state and in the later sections how to perform hybrid BSM in this architecture. In the following section, the fidelity of the hybrid quantum teleportation is compared with that of the classical teleportation. It shows a clear distinction between quantum and classical channels for the quality of our teleportation. Finally, the schemes of verification of ECSs and of a single-qubit gate on ECSs are proposed toward universal hybrid quantum computing in circuit-QED.

## Results

### A two-cavity and three-qubit (2C3Q) architecture: reduction of self-Kerr effects

Before we present the details of hybrid teleportation, let us discuss one of the major barriers to realising complex protocols involving ECS, namely the ‘self-Kerr effect’. The nonlinearity of superconducting qubits and their strong interaction with the cavity generate an effective nonlinearity in the cavity which is called self-Kerr. This is often very useful for creating Josephson parametric amplifiers^67^ and nonclassical microwaves^68^,^69^ in circuit-QED. In the case of ECS which involve two cavities, the qubit nonlinearity can also induce additional cross-cavity nonlinearities, but the case for ECS can be simply demonstrated with one cavity cat-states. These Kerr-type nonlinearities create distortions of the quantum states which beyond a certain level can become a source of error in the quantum information processing protocol. Therefore it is important to find ways to mitigate their strength.

In order to demonstrate the importance of reducing the self-Kerr interaction let us consider the state of the system before the teleportation protocol begins. The qubit *A* that will later hold the information to be teleported is initialised to the ground state |*g*〉_*A*_. The entangled-CV channel is initialised to be in an ECS, written as


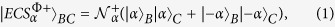


where 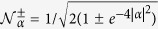 is a normalisation and 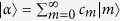 (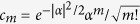). Note that four Bell-type ECSs are defined by 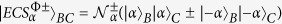 and 
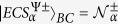

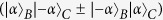
38,39,49.

According to the results in ref. 61, the imperfect preparation of the generated ECS can be however estimated as 96% in 190 ns under realistic defects given by self-Kerr and cross-Kerr effects. It might be ignorable for short-time quantum operation but cannot be avoidable for longer-time quantum information processing like teleportation. For example, although the distribution of the probability amplitudes are kept in coherent states and SCSs, the Wigner functions are distorted under the self-Kerr effect. This is because the rotational speed of relative phases depend on the photon numbers in Fock states.

**Figure 1:**
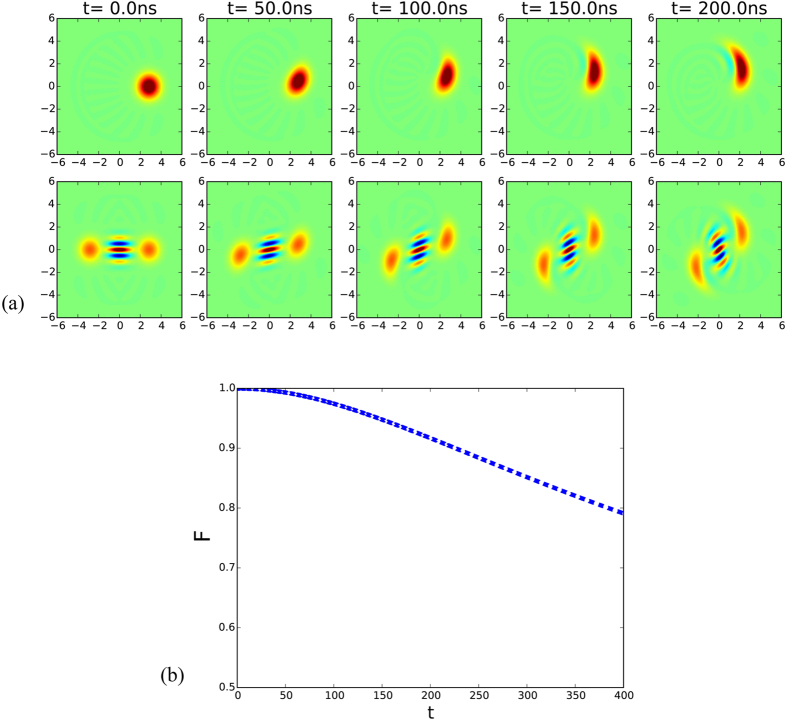
(**a**) The array of Wigner functions show that coherent state |2.0〉_*C*_ and the even SCS 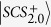 start from *t* = 0 and evolve for *t* = 200 ns under the Hamiltonian 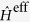 given by Eq. (2) (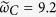 GHz and *K* = −66.7 kHz). The shapes of the states in phase space are distorted and the maximum fidelity at *t* is given by 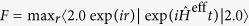 upto *t* = 400 ns in (**b**).

In order to estimate a single-photon Kerr effect^70^,^71^, we examine the Kerr frequency *K* in the effective Hamiltonian for cavity photons given by


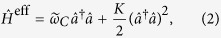


where 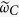 is a cavity frequency in dressed states and 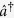 is a creating operator for photons, see the derivation of the Hamiltonian Eq. (3.24) in ref. 72. As an example, we examine how the self-Kerr term affects different a CV qubit. Figure 1(a) shows that coherent states and SCSs are squeezed in phase space and the fidelity between ideal and distorted states is depicted for evolution time *t* = 200 ns. The fidelity *F* between the ideal and self-Kerr distorted states indicates the defects of CV qubit states under the self-Kerr term in Eq. (2) as shown in Fig. 1(b).

However, if the self-Kerr effect can be much reduced over our proposed teleportation scheme, the quality of the ECS channel and the fidelity of the outcomes will be well-enough during the total operation time reported very recently as near 1 ms^73^. In addition, the method of selective number-dependent arbitrary phase gates, called SNAP gate, has been demonstrated in coherent states and could make additional reduction of the self-Kerr defects dynamically in the form of SCSs^74^. In contrast our method yields a reduction of self-Kerr by design and requires no further state manipulation.

**Figure 2:**
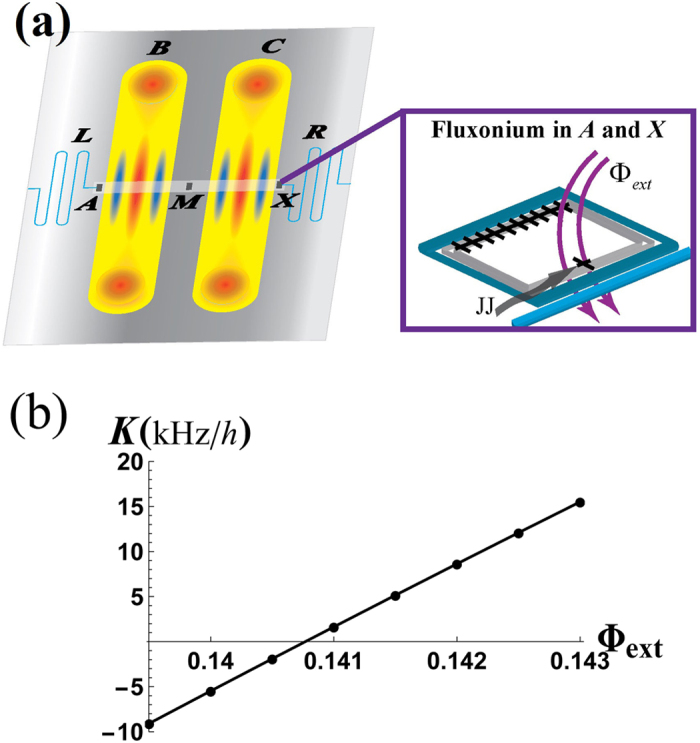
(**a**) An illustration of the architecture of a cavity-QED system named a two-cavity and three-qubit (2C3Q) architecture. The two high-Q cavities (*B* and *C*) posses an ECS and the outer readout resonators (*L* and *R*) are used for measurement of qubits and cavity fields. If the outer qubits (*A* and *X*) are fluxonium^75^,^76^,^77^, the self-Kerr distortion on the cavities might be reducible by an appropriate external flux Φ_*ext*_ (JJ: Josephson-Junction). (**b**) It shows that the self-Kerr effect *K* of a cavity almost linearly changes with respect to the tunable external flux Φ_*ext*_ in a fluxonium.

Figure 2 is depicted for the proof-of-concept that the effective self-Kerr effect in a cavity alters due to external flux Φ_*ext*_ through a fluxonium^75^,^76^,^77^. It has been known that the nonlinearity in the transmon qubit *M* induces the anharmonicity in the energy levels of cavity *B* and *C*^78^,^79^. In the inset of Fig. 2(a), the outer fluxonium qubits (*A* and *X*) parameters are found such that they combine with the transmon to reduce the self-Kerr nonlinearity in a cavity. This in turn will prevent unwanted state-distortion and enhance the total operation time. For fluxonium it is possible to tune the shape of the potential term in its Hamiltonian by applying external magnetic flux perpendicular to the device. This changes the fluxonium spectrum and coupling to the cavity and effectively counter-balances the anharmonicity of the cavity levels induced by the transmon qubit *M*. Thus, the 2C3Q architecture can significantly diminish the induced self-Kerr distortion of the cavity state. The detail calculation is given in the Methods Section.

### Teleportation protocol in the 2C3Q architecture

**Figure 3:**
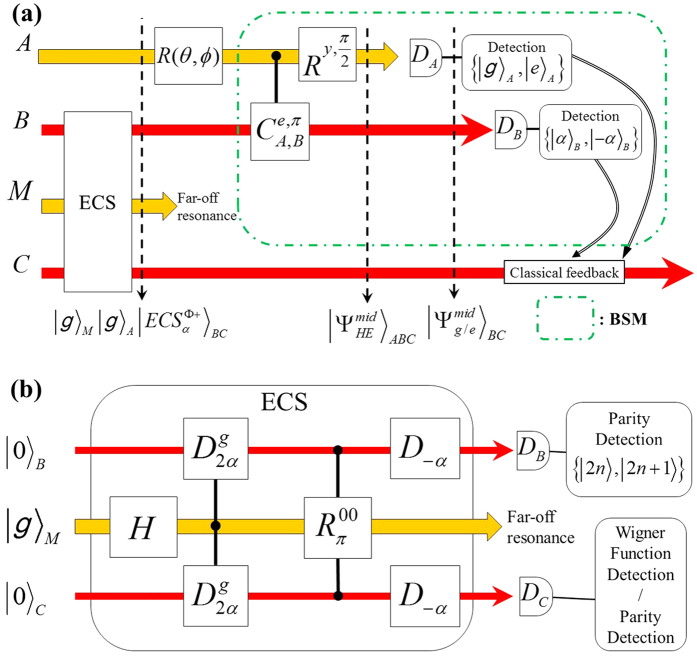
(**a**) Schematics of hybrid teleportation from an unknown superconducting state in *A* to a CV cavity field in *C*. The states of *A* and *M* are superconducting qubits in orange lines while that of *B* and *C* are cavity fields in red lines. The transmon qubit *M* is used for preparation of a ECS and decoupled enough cavity *C* from *B*. The part of implementing the BSM scheme has been demonstrated very recently in^80^. (**b**) Circuit diagram for creating and verifying an ECS^61^. 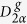 is a conditional displacement with 2*α* while *D*_−*α*_ does unconditional one with −*α*. 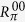 makes a *π*-flip operation of the qubit *M* at a vacuum state in modes *B* and *C. D*_*B*_ and *D*_*C*_ indicate detectors for cavity states. The final detection schemes are explained in the section of the verification scheme for ECSs.

We briefly describe how to implement this hybrid teleportation in circuit-QED. As similar to the DV-qubit teleportation, we begin with the unknown qubit given by |*ψ*〉_*A*_ in a discretized two-level state, represented by ground and excited states of a superconducting qubit (|*g*〉 and |*e*〉). As shown in Fig. 3, before the protocol begins it is prepared in the superconducting state of |*g*〉_*M*_ with two vacuum states in modes *B* and *C*. For preparation of an arbitrary qubit in *A*, a single-qubit operation *R*(*θ*, *ϕ*) is applied on |*g*〉_*A*_ given by


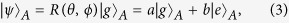


where 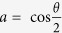 and 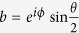. In the preparation stage, it is assumed that the transmon qubit frequency is far off from the cavity resonances to avoid/reduce a direct cross talk between two cavity fields^61^. Based on the implementation scheme in^61^, several sequential gates, see Fig. 3(b), can build the ECS channel in cavities *B* and *C*. In details, after a Hadamard operation on qubit *M*, two conditional displacement operations 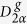 create entanglement among the qubit *M* and two cavity fields. Then, a conditional qubit rotation 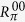 disentangles the cavity-state channel from the qubit and the entangled CV state becomes the form of a maximally entangled state proportional to |0〉_*B*_|0〉_*C*_ + |2*α*〉_*B*_|2*α*〉_*C*_. After the unconditional displacement operation *D*_−*α*_, the outcome state is finally given by 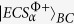.

The total initial state is given by


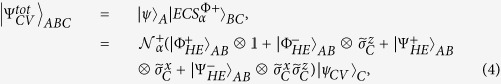


where the hybrid entangled states are


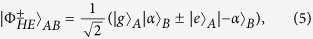



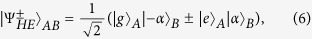


and


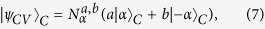


for 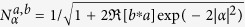 (b^*^: a conjugate of *b*). In particular, even Schrödinger cat is given by 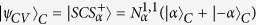 for *a* = *b* while odd Schrödinger cat is given by 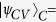 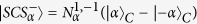 for *a* = −*b*. Then, hybrid BSM is now applied on the state in Eq. (4).

### Hybrid BSM in superconducting and cavity qubits

The hybrid BSM projects the state with the measurement set of 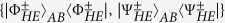 known as a joint measurement between *A* and *B*. To perform the hybrid version of BSM on *A* and *B*, shown in a green box in Fig. 3, two operations are firstly required such as a conditional phase gate between the superconducting qubit *A* and the cavity state *B* as well as a single-qubit rotation in *A*. An entangling gate between *A* and *B* is given by a generalized conditional phase gate *C*^*e*,*φ*^ written by


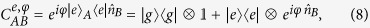


where 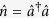, and 
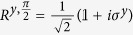
. After the single-qubit operation 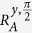 and *φ* = *π*, the total state is equal to


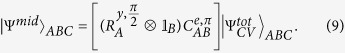


Note that the operation of 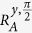 transfers 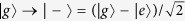 and 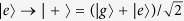. The combination of these operations 
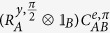
 makes the Bell states, written as 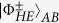 and 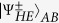 in Eq. (4), into four product states such as |*g*〉_*A*_|±*α*〉_*B*_ and |*e*〉_*A*_|±*α*〉_*B*_.

What Alice needs is now sequential detections on the state of *A* and *B* in the basis sets of {|*g*〉_*A*_, |*e*〉_*A*_} and {|*α*〉_*B*_, |−*α*〉_*B*_} through the low-Q resonator *L*. In Fig. 3(a), two measurements are independently performed in the superconducting qubit ({|*g*〉_*A*_, |*e*〉_*A*_}) first and the cavity field ({|*α*〉_*B*_, |−*α*〉_*B*_}) later. After reading the qubit state in *A* is |*g*〉_*A*_ or |*e*〉_*A*_, the CV-qubit measurement can be performed in {|*α*〉_*B*_, |−*α*〉_*B*_} by recycling the superconducting state collapsed in *A*. A similar measurement technique has been very recently demonstrated in ref. 80. An extra displacement operation *D*(*α*) on |±*α*〉_*B*_ could bring the better distinguishability of the CV state (|2*α*〉_*B*_ and |0〉_*B*_) because its minimum requirement is to identify a vacuum state |0〉_*B*_ conclusively. Once the qubit- and cavity-state measurements are at the level of single-shot measurement with high fidelity, the success probability of the hybrid BSM will be 1/4 in each outcome for *α* ≫ 1, same as for conventional DV teleportation. This is due to the orthogonality of four measurement outcomes in the BSM. In contrast for a non-orthogonal basis measurement a probability smaller (or bigger) than 1/4 for small *α* might occur.

When Alice announces measurement outcomes, Bob obtains the teleported CV state as a generalized SCS. For example, one of the four teleported states is given by 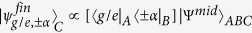 such that


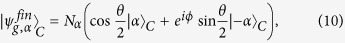


and 
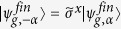
, 
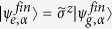
, and 
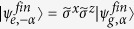
 (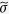 is a pseudo-Pauli operator for |*α*〉 = |0〉 and |−*α*〉 = |1〉). Note that the final result clearly shows the CV version of the original qubit upto pseudo-Pauli operators with 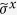 and 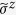. To confirm the sucessful teleported state in mode *C*, the qubit *X* and the most right low-Q resonator *R* will be used for performing a Wigner function plot of the cavity state. Additionally, the unknown superconducting qubit state can be recovered in CV qubit in mode *C* through the pseudo Pauli operator, which can be performed by a qcMAP gate with the superconducting qubit *X*^61^.

### Fidelity of hybrid teleportation

To claim that our hybrid teleportation has been performed through a nonclassical channel, we need to show that the hybrid teleportation fidelity exceeds a threshold value which is achievable with a classically correlated CV state (see details in the Methods section). The teleportation fidelity is determined by the quantumness of the channel state in a teleportation scheme when the BSM is ideal. If the channel suffers decoherence before the BSM, it should be described in mixed states. Based on the criteria of successful quantum teleportation in DV qubits, the average fidelity of a teleported state needs in theory to be higher than 2/3 to claim that a truely quantum channel is used. This is because maximally correlated classical states 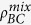 can be used for performing classical teleportation only up to the average fidelity 2/3^81^ (see details in the Section Methods). Full CV teleportation, however, proposes the different threshold of 1/2 for claiming the nonclassicality of a teleportation channel^17^,^18^,^19^. The reason is that an ideal classical channel, namely two coherent states (|*α*〉_*B*_|*α*〉_*C*_), produces the teleportation fidelity 1/2.

In our hybrid teleportation, the comparison of teleportation protocols are more complex because the quantity of the fidelity relies on the degree of decoherence as well as the nonorthogonality given by the initial size of *α* of the channel state. For comparison with the fidelity of hybrid quantum teleportation, we use the fidelity of DV classical teleportation 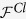 with respect to the angle *θ* in the unknown state given by 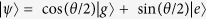 for *ϕ* = 0. Thus, to claim that our CV channel is a nonclassical (or quantum) channel, the fidelity of hybrid teleportation 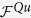 should be better than 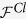 with parameter *α*. For example, we compare the fidelity of hybrid teleportation with that of classical teleportation in the outcome of |*g*〉_*A*_|*α*〉_*B*_ and explain which experimental condition would show a clear distinction between quantum and classical cases.

Because the initial state is in DVs and the teleported state is in CV in our teleportation, we define the teleportation fidelity between a teleported state 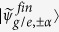 and an expected CV state 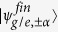 given by


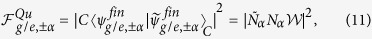


where 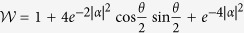. For example, if Alice obtains the outcome state |*g*〉_*A*_|*α*〉_*B*_, the teleported state is given by 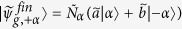 for 
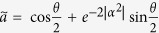
 and 
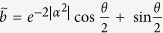
.

**Figure 4:**
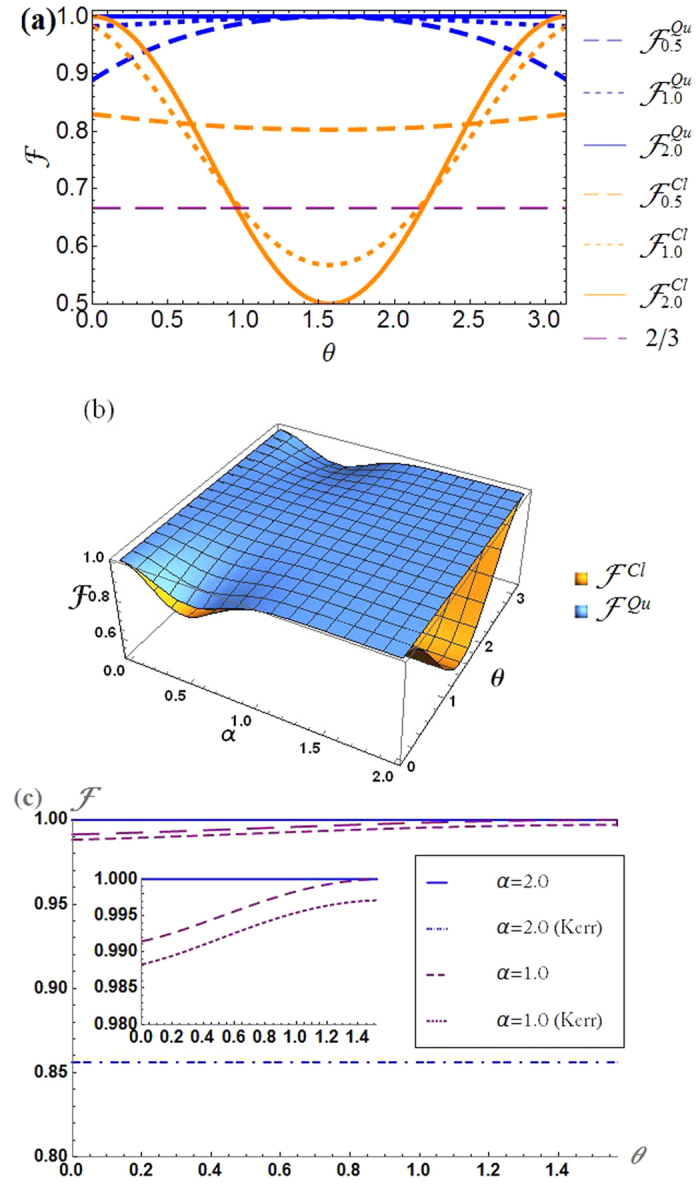
Teleportation fidelities 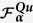 in Eq. (11) and 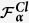 in Eq. (20) for *ϕ* = 0 and |*g*〉_*A*_. (**a**) The orange curve shows that the classical teleportation fidelity cannot excess 1/2 for *θ* = *π*/2 with 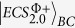 while the fidelity of quantum teleportation is always 1. (**b**) 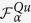 (blue surface) is always better than 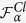 with respect to *α* and *θ*. For example, 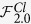 is approximately equal to the fidelity of classical DV teleportation given by 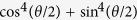. (**c**) The last figure shows that the teleportation fidelity dramatically decreases when we take into account the self-Kerr distortion. The curves are fidelities of quantum cases with/without *K* = −66.7 kHz at *t* = 50 ns in Eq. (2) and the teleported state of *α* = 2.0 suffers more distortion due to the contribution of larger Fock states while the state with small *α* are more robust against self-Kerr effects.

As shown in Fig. 4(a), the fidelity of the hybrid quantum teleportation needs to meet the criteria of classical teleportation fidelity 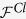 (orange lines) and the details are given by the Method section. For large *α*, the curves show that 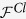 is far less than 1 while 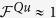. In particular, the value of 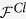 becomes 1/2 at around *θ* = *π*/2^18^,^19^. The reason of 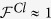 for large *α* with *θ* = 0 is that the classical teleportation also works well if the unknown state is in |*g*〉_*A*_ or |*e*〉_*A*_ as a classical bit. If *α* ≈ 0, 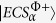 and 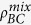 both become a vacuum and two fidelities reaches 1. In Fig. 4(b), the hybrid teleportation fidelity 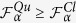 for fixed *α* overall.

Interestingly, for *θ* = 0 (or *π*) and *α* ≈ 0.5, the hybrid teleportation fidelity is far less than 1 because of the effect of the nonorthogonal measurement in {|*α*〉_*B*_, |−*α*〉_*B*_}. However, the fidelity of hybrid quantum teleportation is always the unity (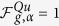) for *θ* = *π*/2 and any size of *α* because the measurement outcomes of |*g*〉_*A*_|*α*〉_*B*_ and |*g*〉_*A*_|−*α*〉_*B*_ bring the identical outcome as 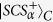. Thus, the issue of nonorthogonal measurement given by small *α* does not affect on the fidelity at *θ* = *π*/2. Therefore, this hybrid quantum teleportation will be able to show a clear advantage from the equally superposed input state |±〉_*A*_ to be teleported in even/odd Schrödinger cat states while |*g*〉_*A*_ and |*e*〉_*A*_ give the same amount of the fidelities for both classical and quantum teleportation. Therefore 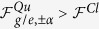 and it is shown that the hybrid teleportation is performed through a nonclassical channel.

Figure 4(c) shows the fact that the self-Kerr effect is harmful to achieve hybrid teleportation with high fidelity because the fidelity dramatically decreases between |*ψ*^*fin*^〉 and 
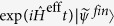
 when we take into account the self-Kerr distortion. The curves are the fidelities of quantum cases with/without *K* = −66.7 kHz upto *t* = 50 ns in Eq. (2) and the case of *α* = 2.0 suffers more distortion due to the contribution of larger Fock states while the fidelity less reduced with *α* = 1.0 under the same condition.

### Verification scheme for ECSs

We here propose a verification scheme of ECSs can be performed by measuring cavity fields in the same architecture shown in Fig. 2 and this scheme will provide us an efficient method of quantum state tomography for specific entangled CV states. Because CV states in principle have infinite dimension, this brings a difficulty to perform a conventional quantum tomography for CV states for example performing a measurement of the amplitudes in the number state basis. However, an ECS can be verified with two sets of measurement schemes if the orthogonality between |*α*〉 and |−*α*〉 are large enough, e.g. *α* > 1.5. As shown in the Methods section below, the basic idea of our verification scheme is inspired from a stabilizer formalism on a Bell state.

To measure the amount of entanglement on a ECS, a Bell-type nonlocality test can be used by observing Wigner functions of two cavity states^61^. However, it does not prove that the prepared state is the desired even ECS although the nonlocality test guarantees the maximal entanglement of two CV qubits. Thus, an efficient scheme of verification on a prepared CV state is a different approach from that how much the amount of entanglement the state has.

As shown in Fig. 3(a), if 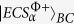 is prepared in the cavities of *B* and *C*, one can independently measure the cavity states connected to outer superconducting qubits (*A* and *X*) and two outer resonators (*L* and *R*). Two measurement schemes are used: one is a measurement setup in the basis-state set {|*α*〉_*B*_, |−*α*〉_*B*_} and the other is a parity measurement of CV states in mode *C*. Alternatively, a measurement of the Wigner function may also be considered.

Two independent detections are first performed in the basis-state set {|*α*〉, |−*α*〉} in both modes *B* and *C*. This measurement results can provide the expectation value of 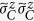 on 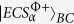. It can show that the correlated measurement outcomes of two cavity states such as |*α*〉_*B*_|*α*〉_*C*_ or |−*α*〉_*B*_|−*α*〉_*C*_ if |*α*〉 is sufficiently orthogonal to |−*α*〉. As we mentioned in the scheme of the hybrid BSM in Results section, an additional displacement operation might give an easier measurement scheme given by detecting a vacuum state |0〉 conclusively. Thus, the outcomes of two cavity states are always identical in both modes *B* and *C* if the prepared state is 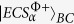.

Even if the outcomes are identical in the 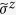 measurement, it does not however provide sufficient information for the verification of the CV state because the perfect correlation might come from the classically correlated state 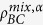, see Eq. (19), but not the quantum correlated state 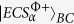. To distinguish them, a parity measurement is required on one (or both) cavity field(s) as the pseudo Pauli-*x* measurement. This parity measurement scheme on cavity states has been already tested in the experiment of superconducting circuits through a superconducting qubit^82^. For example, the parity measurement in mode *C* is equivalent to project the state of *C* onto the basis-state set of even/odd SCSs and forces to collapse the cavity state of *B* into an even/odd SCS such that


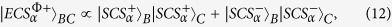


where even SCSs can be also represented by the sum of even photon-number states 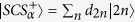 and odd SCSs do that of odd photon-number states 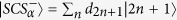. In other words, the outcome state in mode *B* has to have a fringe pattern in Wigner function distribution in *B* after the parity measurement in *C*^82^ because the outcome of even (odd) parity brings an even (odd) SCS in mode *B*. Thus, the perfect correlation of the parity measurement outcomes occurs only if the prepared state is the ECS. On the other hand, this parity measurement on one of the classically correlated state in Eq. (19) provides a fully mixed state and no fringe patten in mode *B* given by


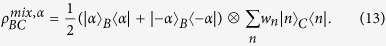


Therefore, two measurement sets of pseudo Pauli operators on CV qubits can verify the state of 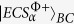 in two cavities.

### Single CV-qubit gate on ECSs

A set of one- and two-qubit gates are essential gates for universal quantum computing. As explained in the hybrid teleportation scheme, the final state before classical feed-forward is indeed a excellent candidate for a single CV-qubit operation (see below in Eq. (10)) although it destroys the ECS. We here present a different and deterministic scheme of a pseudo Pauli-*x* gate 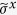 on a CV-qubit of the ECS in the 2C3Q architecture. The key idea is based on a non-destructive syndrome measurement by entangling and measuring outer ancillary qubits. After the series of performing 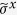, two CV-qubit entangled states are altered without destroying the CV-qubits (e.g., 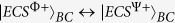). Alternatively, it is useful for robust CV quantum information processing in circuit-QED because a single-qubit error in entangled CV qubits can be monitored by entangling and measuring outer ancillary qubits.

**Figure 5:**
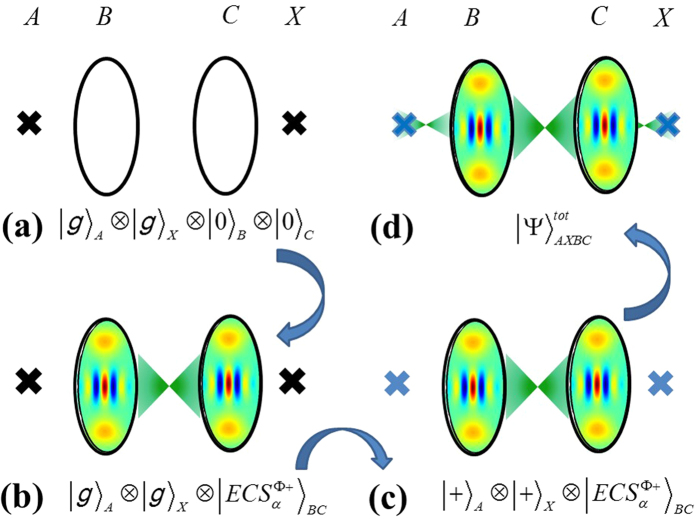
(**a**) The initial state is prepared in |*g*〉_*A*_|*g*〉_*X*_|0〉_*B*_|0〉_*C*_. (**b**) The ECS is created by the scheme in Fig. 3. After two Hadamard operations in superconducting qubits of *A* and *X* in (**c**), two entangling gates between the superconducting qubit *A* (*X*) and the CV qubit *B* (*C*) given by 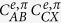 to create the four-partite entangled state in (**d**).

Since the verification scheme of ECSs guarantees the good quality of a ECS preparation, CV quantum information processing is indeed viable in superconducting circuits. In DV qubits, a Pauli-*x* gate is known as the quantum NOT gate performing an operation between *a*|0〉 + *b*|1〉 ↔ *b*|0〉 + *a*|1〉 and it is equivalent to the operation *a*|*α*〉 + *b*|−*α*〉 ↔ *b*|*α*〉 + *a*|−*α*〉. The CV scheme is a repeat-until-success-type single-qubit CV gate performed by repetition of entangling and measuring outer superconducting qubits within decoherence time. This protocol is similar to the above verification scheme because both rely on the stabilizer formalism given in Eq. (22). The key difference from the verification is that it uses the quantum nondemolition measurement where ancillary (outer) qubits are additionally entangled with the ECS. The outer qubits provide information about the CV qubits without destroying the ECSs. Thus, this scheme can be in general applicable for the 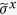 operation in multipartite CV entangled states. For realising a syndrome detection without a CV state collapse we could create a four-partite hybrid entangled state between the outer superconducting qubits (*A*, *X*) and the ECS in *B* and *C*, see the Figs 2(a) and 5. There, the parity of the outer superconducting qubits is detected in the measurement set of {|*g*〉, |*e*〉}.

As the schematic protocol is depicted for building a specific four-qubit entangled state in Fig. 5, (a) we begin with four separable states in two superconducting qubits and two cavities such as |*g*〉_*A*_|*g*〉_*X*_|0〉_*B*_|0〉_*C*_. (b) The method of creating the ECS is performed by Fig. 3 and the state is prepared in 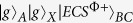. Note that we omit the superconducting qubit *M* between two cavities here because the qubit is far-detuned from cavity frequency and does not participate in the operations after creating the ECS. (c) A Hadamard operation are performed in the superconducting qubits resulting in 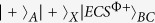 (e.g., 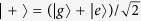). (d) After the entangling operation of 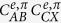, the four-partite entangled state is equal to


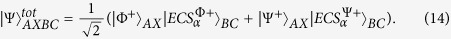


This state is known as a four-qubit Greenberger-Horne-Zeilinger state in two superconducting qubits and two cavity states because the state in Eq. (14) is rewritten by


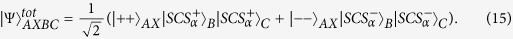


Thus, the measurement outcomes of qubits *A* and *X* in {|*g*〉, |*e*〉} determine the two-cavity state in 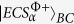 or 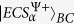 in Eq. (14) while the measurements in {|+〉, |−〉} brings a product state of two SCSs in modes *B* and *C* in Eq. (15).

For the repeat-until-success protocol, if the measurement outcomes are |*g*〉 (or |*e*〉) in both superconducting qubits, the two-cavity state is still kept in 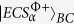 while the cavity state is successfully collapsed into the desired state of 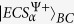 with different measurement outcomes in modes *A* and *X*. Thus, the former outcome becomes the state depicted in Fig. 5(b) and two entangling gates are performed again between *A* and *B* as well as *C* and *X* within coherence time in Fig. 5(c,d). Finally, one of the cavity states is conditionally flipped between |*α*〉 and |−*α*〉 on 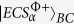.

## Conclusion

We propose a teleportation scheme from a superconducting DV qubit to a microwave CV qubit in superconducting circuits. The proposed architecture of two cavities and three superconducting qubits is currently within reach with realistic parameters in the state-of-the-art platform of circuit-QED. The unknown state in a superconducting qubit is teleported via the ECS created between two cavities. The hybrid Bell measurement encodes the quantum information in the unknown qubit into a CV qubit in a cavity state. The teleportation fidelity in the hybrid scheme can confirm that the ECS channel is a nonclassical resource with respect to the size of *α*. The same architecture is also beneficial for other CV quantum information processing for the schemes of verification and error-correction in the ECS channel. Finally, by using two different superconducting qubits, we present a way of mitigating the adverse effect the Kerr distortion and opening the way to the possible realisation of complex teleportation-based protocols.

Toward hybrid measurement-based quantum computing in circuit-QED, the capability of building a two CV-qubit gate between two cavities might be of essence in addition to single-qubit gates in superconducting circuits^83^. For example, linear four-qubit hybrid cluster states will give a strength of one- and two-qubit gates which has been investigated in photonic measurement-based quantum computation^84^. For example, the outcomes of the teleported state before the classical feedback contain one of the single-qubit gates (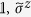, 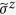, 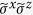) probabilistically while a deterministic gate for 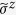 needs to transform the CV state from *N*_*α*_(*a*|*α*〉 + *b*|−*α*〉) to *N*_*α*_(*a*|*α*〉 − *b*|−*α*〉). This 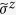 gate could be viable by performing a photon-shifting gate 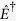 proposed in^85^ based on the setup in Fig. 2(a). In details, since 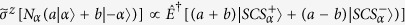, 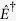 is equivalent to 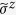 because 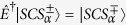 and 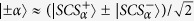 for large *α*. A two-CV-qubit gate might be also feasible through measuring DV superconducting qubits opening the way towards universal quantum computing. To overcome errors in both superconducting and cavity qubits, we may need to build logical hybrid qubits with logical cluster states or to entangle higher-dimensional CV-qudits with superconducting qubits^46^,^57^,^58^,^86^. For creating multi-partite ECSs^87^, cross-Kerr interaction could be used in the multiple-cavity architecture joined by mediating qubits in order to keep the capability of CV-qubit operations in a dispersive regime.

## Methods

### Hamiltonian for the 2C3Q architecture and reduction of the Kerr-effect

To demonstrate the principle of the reducing method in superconducting circuits, we examine a half of the 2C3Q system in Fig. 2(a) due to its symmetry of the full architecture and a cavity is sandwiched between a fluxonium and a transmon given by the Hamiltonian


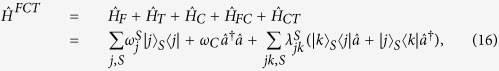


for *S* = *F*, *T* and *j* < *k* (*ħ* = 1). The pseudo-photonic eigenstates and eigenvalues given by transmon and fluxonium ground states with 0, 1, and 2 photons in the cavity mode can be calculated given by *j*, *k* = 0, 1, 2. For example, we obtain *K* ≈ −66.7 kHz in a system of a transmon and a cavity with the absence of a fluxonium (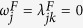) while *K* ≈ 170.8 kHz is given by the system of a fluxonium and cavity with 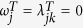. If we include both superconducting qubits with the cavity commonly connected, the self-Kerr effect *K* can be reduced. Therefore, if |Φ_*ext*_| = 0.141, the self-Kerr effect reduces *K* ≈ 1.64 kHz (from −66.7 kHz without fluxonium) and the architecture of the fluxonium-cavity-transmon can tune the self-Kerr effect in a cavity by design in circuit-QED systems.

For demonstration of the self-Kerr effect in Fig. 2(b), we set up realistic parameters such that the cavity frequency as *ω*^*C*^ = 9.2 GHz and the transmon energy levels 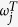 and coupling strengths 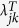 are given by 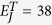 GHz and 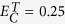 GHz (see details in ref. 78 and 79) while the fluxonium parameters are 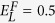 GHz, 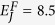 GHz and 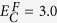 GHz^75^,^76^,^77^. For example, if |Φ_*ext*_| = 0.141, 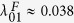 GHz, 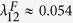 GHz, and 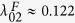 GHz^75^,^76^,^77^ while 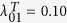 GHz, 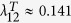 GHz, and 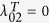.

### DV quantum and classical teleportation

Let us first briefly describe quantum teleportation in DV qubits. An unknown qubit state is given in 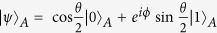 and Alice and Bob have already shared one of the Bell states in modes *B* and *C* such as 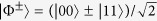 and 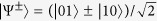 and we assume that the total state is initially prepared with |Φ^+^〉_*BC*_ given by


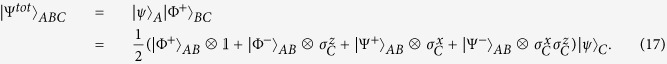


This mathematical representation implies what are the teleported state dependent on the measurement outcomes of the BSM. After Alice performs a BSM in 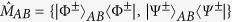, she announces measurement outcomes to Bob to reconstruct the unknown state by applying one of four single-qubit operations (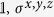).

A maximally correlated classical state can be also used for a teleportation channel given by


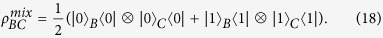


The BSM is performed as 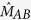 and the fidelity of the DV teleportation with the unknown state |*ψ*〉_*A*_ (*ϕ* = 0) is represented by 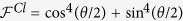, which is approximately equivalent to the classical CV teleportation (see the orange solid line in Fig. 4(a)). The details of DV classical teleportation for fidelity is described in^88^.

### Fidelity of hybrid classical teleportation

A classical CV channel is given by


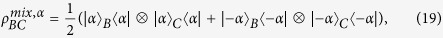


which can be understood as the state suffering a decoherence from the ideal ECS channel. We now perform a joint measurement between the unknown DV qubit in *A* and the CV qubit in *B*. Thus, the fidelity between 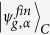 and a classically teleported state 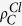 through 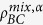 is given by


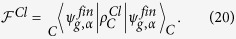


We here examine the fidelity characteristics with *ϕ* = 0 and the outcome of |*g*〉_*A*_|*α*〉_*B*_. For example, the classically teleported state has lost the coherence of the unknown state and is given by


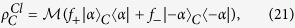


where 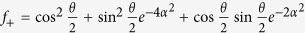 and 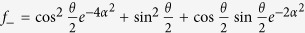. Note that 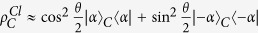 for *α* > 1.


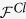
 is approximately equal to 
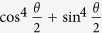
 for large *α*, which is also obtained by DV classical teleportation with |*ψ*〉_*A*_ and 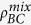. In contrast to DV- and CV-only teleportations, the fidelities decrease not only with the decoherence of the ECS channel but also with the size of *α* in the coherent-state representation. For example, for small *α*, 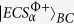 tends to behave similar to two vacuum states but still maintains the superposition between |*α*〉|*α*〉 and |−*α*〉|−*α*〉.

### Syndrome measurement and stabilizer formalism for DV qubits

To verify a Bell state 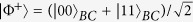, one only needs to obtain the expectation values of 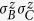 and 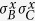 on |Φ^+^〉 because |Φ^+^〉 is the only state which always provides +1 eigenvalue for the two sets of Pauli operators given by


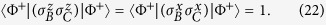


The analogue of pseudo single-qubit gate in Results section begins with |Φ^+^〉_*BC*_ and |+〉_*A*_|+〉_*X*_ and we want to obtain a *σ*^*x*^-operated |Φ^+^〉_*BC*_ without destroying qubits *B* and *C*, which is equivalent to 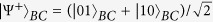. After two-qubit gates (i.e., CNOT gate) are preformed between *A* and *B* as well as *C* and *X*, a four-qubit GHZ state 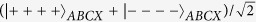 is created as similar to 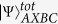 in Eq. (14). If two single qubits are measured in 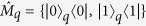 for *q* = *A*, *X*, the final state is either |Φ^+^〉_*BC*_ with the same outcomes or |Ψ^+^〉_*BC*_ with different outcomes in *A* and *X*.

## Additional Information

**How to cite this article**: Joo, J. and Ginossar, E. Efficient scheme for hybrid teleportation via entangled coherent states in circuit quantum electrodynamics. *Sci. Rep.* **6**, 26338; doi: 10.1038/srep26338 (2016).
